# Computing Topological Invariants of Deep Neural Networks

**DOI:** 10.1155/2022/9051908

**Published:** 2022-10-07

**Authors:** Xiujun Zhang, Nazeran Idrees, Salma Kanwal, Muhammad Jawwad Saif, Fatima Saeed

**Affiliations:** ^1^School of Computer Science, Chengdu University, Chengdu, China; ^2^Department of Mathematics, Government College University Faisalabad, Faisalabad 38000, Pakistan; ^3^Department of Mathematics, Lahore College for Women University, Lahore 54000, Pakistan; ^4^Department of Applied Chemistry, Government College University Faisalabad, Faisalabad 38000, Pakistan

## Abstract

A deep neural network has multiple layers to learn more complex patterns and is built to simulate the activity of the human brain. Currently, it provides the best solutions to many problems in image recognition, speech recognition, and natural language processing. The present study deals with the topological properties of deep neural networks. The topological index is a numeric quantity associated to the connectivity of the network and is correlated to the efficiency and accuracy of the output of the network. Different degree-related topological indices such as Zagreb index, Randic index, atom-bond connectivity index, geometric-arithmetic index, forgotten index, multiple Zagreb indices, and hyper-Zagreb index of deep neural network with a finite number of hidden layers are computed in this study.

## 1. Introduction

Neural networks are not only studied in artificial intelligence but also have got great applications in intrusion detection systems, image processing, localization, medicine, and chemical and environmental sciences [1–3]. Neural networks are used to model and learn complex and nonlinear relationships, which is very important in real life because many of the relationships of inputs and outputs are nonlinear and complex. Artificial neural networks are the backbone of robotics, defense technology, and neural chemistry. Neural networks are not only being widely used as a tool for predictive analysis but also trained successfully to model processes including crystallization, adsorption, distillation, gasification, dry reforming, and filtration in neural chemistry [4–8].

The topological index associates a unique number to a graph or network, which provides correlation with the physiochemical properties of the network. Degree-based topological index depends upon the connectivity of the network. The first degree-based topological index, called the Randić index, was formulated by Milan Randić [9] while analyzing the boiling point of paraffin. Over the last three decades, hundreds of topological indices have been formulated by researchers, which are helpful in studying the different properties of chemical graphs like reactivity, stability, boiling point, enthalpy of formation, and Kovat's constant and inherits physical properties of materials such as stress, elasticity, strain, mechanical strength, and many others.

Bollobás and Erdős [10] introduced the general Randić index given by equation (1). The first and second Zagreb indices were introduced by Gutman and Trinajstić [11] in 1972, which appeared during the analysis of *π*-electron energy of atoms. The multiplicative version of these Zagreb indices (the first multiplicative Zagreb index and the second multiplicative Zagreb index) of a graph were formulated by Ghorbani and Azimi [12]. Shirdel et al. [13] introduced a new version of Zagreb indices named as the hyper-Zagreb index. The widely used atom-bond connectivity (ABC) index is introduced by Estrada et al. [14]. Zhou and Trinajstić [15] gave the idea of the sum-connectivity index (SCI). The geometric-arithmetic index was introduced by Vukičević and Furtula [16]. Javaid et al. [17] investigated the degree-based topological indices for the probabilistic neural networks in 2017. Topological indices for multilayered probabilistic neural networks and recurrent neural networks have also been computed recently [18–21]. For more work-related to computation and bounds of topological indices, see [22–29].

Consider a graph *G* having a set of nodes *V*and a set of edges *E*. Degree of a node *v*, denoted by *d*_*v*_, is the number of nodes connected to *v* via an edge. A degree-based topological indices of a graph *G* are defined as follows:

Randić index

(1) $$\begin{array}{c} \chi \left(G\right)=\underset{uv\in E}{\sum }\left(1/\sqrt{d_{u}d_{v}}\right)\text{.} \end{array}$$

General Randić index

(2) $$\begin{array}{c} R_{\alpha }\left(G\right)=\underset{uv\in E}{\sum }{\left(d_{u}d_{v}\right)}^{\alpha }\text{.} \end{array}$$

First Zagreb index

(3) $$\begin{array}{c} M_{1}\left(G\right)=\underset{uv\in E}{\sum }\begin{array}{c} \left[d_{u}+d_{v}\right] \end{array}\text{.} \end{array}$$

Second Zagreb index

(4) $$\begin{array}{c} M_{2}\left(G\right)=\underset{uv\in E}{\sum }\begin{array}{c} \left[d_{u}d_{v}\right] \end{array}\text{.} \end{array}$$

First multiple Zagreb index

(5) $$\begin{array}{c} {PM}_{1}\left(G\right)=\underset{uv\in E}{\prod }\left[d_{u}\times d_{v}\right]\text{.} \end{array}$$

Second multiple Zagreb index

(6) $$\begin{array}{c} {PM}_{2}\left(G\right)=\underset{uv\in E}{\prod }\left[d_{u}\times d_{v}\right]\text{.} \end{array}$$

Hyper-Zagreb index

(7) $$\begin{array}{c} HM\left(C\left[m,n\right]\right)=\underset{uv\in E\;}{\sum }{\left(d_{u}{+d}_{v}\right)}^{2}. \end{array}$$

Atom-bond connectivity index

(8) $$\begin{array}{c} ABC\left(G\right)=\underset{uv\in E}{\sum }\sqrt{\left(d_{u}+d_{v}-2/d_{u}d_{v}\right)}\text{.} \end{array}$$

Sum connectivity index

(9) $$\begin{array}{c} SCI\left(G\right)=\underset{uv\in E\;}{\sum }\left(1/\sqrt{d_{u}{+d}_{v}}\right)\text{.} \end{array}$$

Geometric-arithmetic index

(10) $$\begin{array}{c} GA\left(G\right)=\underset{uv\in E}{\sum }\left(2\sqrt{d_{u}d_{v}}/d_{u}+d_{v}\right)\text{.} \end{array}$$

## 2. Methodology

**Figure 1:**
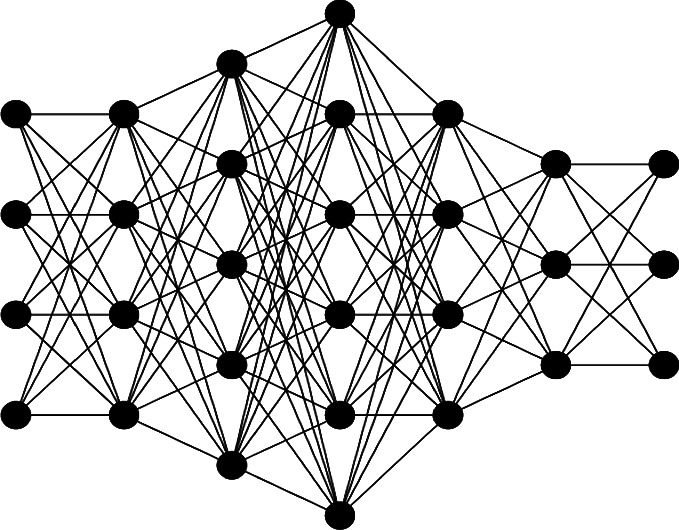
A deep neural network with five hidden layers of DNN (4,4,5,6,4,3,3).

A deep neural network (DNN) can be represented by a graph *Z* : =(*V*, *E*), where *V*denotes the nodes of the network and*E* denotes the set of edges between the nodes. We consider a DNN with an input layer having *M* nodes, *r* hidden layers each layer having *N*_*i*_, *i*=1,2,…, *r* number of nodes such that the first layer has *N*_1_ nodes, the second layer has *N*_2_ nodes, and similarly, the r-th layer has *N*_*r*_ nodes, which can also be expressed as *DNN*(*N*_1_*N*_2_ … *N*_*r*_). The output layer of DNN has *N* nodes. Each node of every layer is connected to all nodes of the next layer. For instance, Figure 1 shows a DNN with an input layer having four nodes, an output layer with three nodes, and five hidden layers.

We first partition the edges of the graph of DNN according to the degree of end vertices of the graph. We analyze the structure of the graph by considering the connectivity of vertices of each layer to the next layer. In DNN, each node of every layer is connected to all nodes of the next layer. This fact is employed to count the degree of each vertex. Consider a deep neural network *DNN*(*N*_1_*N*_2_ … *N*_*r*_). Each node in the input layer has a degree *N*_1_ because every input node is connected to each node of a first hidden layer having *N*_1_ nodes. In the first hidden layer, all node (*N*_1_) has the same degree, i.e., *M*+*N*_2_. Nodes of the second layer have degree *N*_1_+*N*_3_. Similarly, the nodes of *i*-th hidden layer have degree *N*_*i*−1_+*N*_*i*+1_. The nodes of the output layer have degree *N*_*r*_.

We will compute topological indices using the edge partition method. We will classify the edges on basis of degrees of end-nodes of the edges. The number of edges connecting the input layer to the first hidden layer is *N*_1_ , whose end-nodes have degrees *N*_1_ and *M*+*N*_2_. The edges connecting *i*-th hidden layer to *i*+1-st layer have end-nodes having degrees *N*_*i*−1_+*N*_*i*+1_ and *N*_*i*_+*N*_*i*+2_ and the number of such edges is *N*_*i*_*N*_*i*+1_ . Similarly, the *N*_*r*_*N* edges connecting the last hidden layer to the output layer have degrees *N*_*r*−1_+*N* and *N*_*r*_ of end-nodes. These findings are summarized in Table 1 below, which will be further helpful in computing the topological indices.

## 3. Results and Discussions

In this section, we have derived the expressions to compute the topological indices of the deep neural network. These results are related to the connectivity of nodes of DNN.

**Theorem 1 .**

Let *Z*≅*DNN*(*N*_1_*N*_2_ … *N*_*r*_) be a deep neural network. Then the Randić index(*R*_(1/2)_(*Z*)) and general Randić index (*R*_*α*_(*Z*)) of DNN are given as

- $R_{\left(1/2\right)}\left(Z\right)=\left(MN_{1}\right)\sqrt{\left(N_{1}\right)\left(M+N_{2}\right)}+\left(N_{1}N_{2}\right)\sqrt{\left(M+N_{2}\right)\left(N_{1}{+N}_{3}\right)}+\left(\sum_{i=2}^{r-2}N_{i}N_{i+1}{\left(\left(N_{i-1}+N_{i+1}\right)\left(N_{i}+N_{i+2}\right)\right)}^{\left(1/2\right)}\right)+\left(N_{r-1}N_{r}\right)\sqrt{\left(N_{r-2}+N_{r}\right)\left(N_{r-1}+N\right)}+\left(N_{r}N\right)\sqrt{\left(N_{r-1}+N\right)\left(N_{r}\right)}\text{,}$
- *R*_*α*_(*Z*)=(*MN*_1_)((*N*_1_)(*M*+*N*_2_))^*α*^+(*N*_1_*N*_2_)((*M*+*N*_2_)(*N*_1_+*N*_3_))^*α*^+(∑_*i*=2_^*r*−2^*N*_*i*_*N*_*i*+1_((*N*_*i*−1_+*N*_*i*+1_)(*N*_*i*_+*N*_*i*+2_))^*α*^)+(*N*_*r*−1_*N*_*r*_)((*N*_*r*−2_+*N*_*r*_)(*N*_*r*−1_+*N*))^*α*^+(*N*_*r*_*N*)((*N*_*r*−1_+*N*)(*N*_*r*_))^*α*^.

**Proof**

We calculated the degrees of end nodes of every edge for *DNN*(*N*_1_*N*_2_ … *N*_*r*_). By using the definitions and values from Table 1, we get the following results:

(i)
$R_{\left(1/2\right)}\left(Z\right)=\underset{uv\in E}{\sum }\sqrt{d_{u}d_{v}}=\underset{uv\in E\left(N_{1},M+N_{2}\right)}{\sum }\sqrt{d_{u}d_{v}}+\underset{uv\in E\left(M+N_{2},N_{1}{+N}_{3}\right)}{\sum }\sqrt{d_{u}d_{v}}+\underset{uv\in E\left(N_{1}+N_{3},N_{2}+N_{4}\right)}{\sum }\sqrt{d_{u}d_{v}}\ldots \underset{uv\in E\left(N_{r-3}{+N}_{r-1}{,N}_{r-2}{+N}_{r}\right)}{\sum }\sqrt{d_{u}d_{v}}+\underset{uv\in E\left(\left(N_{r-2}+N_{r}N_{r-1}+N\right)\right)}{\sum }\sqrt{d_{u}d_{v}}+\underset{uv\in E\left(N_{r-1}+N,N_{r}\right)}{\sum }\sqrt{d_{u}d_{v}}$

Substituting values from Table 1, we get
  (11) $$\begin{array}{c} R_{\left(1/2\right)}\left(Z\right)=\left(MN_{1}\right)\sqrt{\left(N_{1}\right)\left(M+N_{2}\right)}+\left(N_{1}N_{2}\right)\sqrt{\left(M+N_{2}\right)\left(N_{1}{+N}_{3}\right)}+\left(N_{2}N_{3}\right)\sqrt{\left(N_{1}+N_{3}\right)\left(N_{2}+N_{4}\right),}+\ldots +\left(N_{r-2}N_{r-1}\right)\sqrt{\left(N_{r-3}{+N}_{r-1}\right)\left(N_{r-2}{+N}_{r}\right)}+\left(N_{r-1}N_{r}\right)\sqrt{\left(N_{r-2}+N_{r}\right)\left(N_{r-1}+N\right)}+\left(N_{r}N\right)\sqrt{\left(N_{r-1}+N\right)\left(N_{r}\right)}\text{.} \end{array}$$
This can be expressed as follows:
  (12) $$\begin{array}{c} R_{\left(1/2\right)}\left(Z\right)=\left(MN_{1}\right)\sqrt{\left(N_{1}\right)\left(M+N_{2}\right)}+\left(N_{1}N_{2}\right)\sqrt{\left(M+N_{2}\right)\left(N_{1}{+N}_{3}\right)}+\overset{r-2}{\underset{i=2}{\sum }}N_{i}N_{i+1}{\left(\left(N_{i-1}+N_{i+1}\right)\left(N_{i}+N_{i+2}\right)\right)}^{\left(1/2\right)}+\left(N_{r-1}N_{r}\right)\sqrt{\left(N_{r-2}+N_{r}\right)\left(N_{r-1}+N\right)}+\left(N_{r}N\right)\sqrt{\left(N_{r-1}+N\right)\left(N_{r}\right)}\text{.} \end{array}$$
(ii)
$R_{\alpha }\left(Z\right)=\underset{uv\in E\left(N_{1},M+N_{2}\right)}{\sum }{\left(d_{u}d_{v}\right)}^{\alpha }+\underset{uv\in E\left(M+N_{2},N_{1}{+N}_{3}\right)}{\sum }{\left(d_{u}d_{v}\right)}^{\alpha }+\underset{uv\in E\left(N_{1}+N_{3},N_{2}+N_{4}\right)}{\sum }{\left(d_{u}d_{v}\right)}^{\alpha }+\ldots +\underset{uv\in E\left(N_{r-3}{+N}_{r-1}{,N}_{r-2}{+N}_{r}\right)}{\sum }{\left(d_{u}d_{v}\right)}^{\alpha }+\underset{uv\in E\left(\left(N_{r-2}+N_{r}N_{r-1}+N\right)\right)}{\sum }{\left(d_{u}d_{v}\right)}^{\alpha }+\underset{uv\in E\left(N_{r-1}+N,N_{r}\right)}{\sum }{\left(d_{u}d_{v}\right)}^{\alpha }$

Using Table 1, we get

(13) $$\begin{array}{c} R_{\alpha }\left(Z\right)=\left(MN_{1}\right){\left(\left(N_{1}\right)\left(M+N_{2}\right)\right)}^{\alpha }+\left(N_{1}N_{2}\right){\left(\left(M+N_{2}\right)\left(N_{1}{+N}_{3}\right)\right)}^{\alpha }+\left(N_{2}N_{3}\right){\left(\left(N_{1}+N_{3}\right)\left(N_{2}+N_{4}\right)\right)}^{\alpha }+\ldots +\left(N_{r-2}N_{r-1}\right){\left(\left(N_{r-3}{+N}_{r-1}\right)\left(N_{r-2}{+N}_{r}\right)\right)}^{\alpha }+\left(N_{r-1}N_{r}\right){\left(\left(N_{r-2}+N_{r}\right)\left(N_{r-1}+N\right)\right)}^{\alpha }+\left(N_{r}N\right){\left(\left(N_{r-1}+N\right)\left(N_{r}\right)\right)}^{\alpha }\text{.} \end{array}$$

This can be further summarized as

(14) $$\begin{array}{c} R_{\alpha }\left(Z\right)=\left(MN_{1}\right){\left(\left(N_{1}\right)\left(M+N_{2}\right)\right)}^{\alpha }+\left(N_{1}N_{2}\right){\left(\left(M+N_{2}\right)\left(N_{1}{+N}_{3}\right)\right)}^{\alpha }+\overset{r-2}{\underset{i=2}{\sum }}N_{i}N_{i+1}{\left(\left(N_{i-1}+N_{i+1}\right)\left(N_{i}+N_{i+2}\right)\right)}^{\alpha }+\left(N_{r-1}N_{r}\right){\left(\left(N_{r-2}+N_{r}\right)\left(N_{r-1}+N\right)\right)}^{\alpha }+\left(N_{r}N\right){\left(\left(N_{r-1}+N\right)\left(N_{r}\right)\right)}^{\alpha }\text{.} \end{array}$$

**Theorem 2 .**

Let *Z*≅*DNN*(*N*_1_*N*_2_ … *N*_*r*_) be a deep neural network. Then, first Zagreb index(*M*_1_(*Z*)), second Zagreb index(*M*_2_(*Z*)), first multiplicative Zagreb(*PM*_1_(*Z*)) index, and second multiplicative Zagreb index(*PM*_2_(*Z*)) of DNN are given as follows:

- *M*_1_(*Z*)=(*MN*_1_)(*N*_1_+*M*+*N*_2_)+(*N*_1_*N*_2_)(*M*+*N*_2_+*N*_1_+*N*_3_)+∑_*i*=2_^*r*−2^*N*_*i*_*N*_*i*+1_(*N*_*i*−1_+*N*_*i*+1_+*N*_*i*_+*N*_*i*+2_)+(*N*_*r*−1_*N*_*r*_)(*N*_*r*−2_+*N*_*r*_+*N*_*r*−1_+*N*)+(*N*_*r*_*N*)(*N*_*r*−1_+*N*+*N*_*r*_)
- *M*_2_(*Z*)=(*MN*_1_)[(*N*_1_)(*M*+*N*_2_)]+(*N*_1_*N*_2_)[(*M*+*N*_2_)(*N*_1_+*N*_3_)]+∑_*i*=2_^*r*−2^*N*_*i*_*N*_*i*+1_(*N*_*i*−1_+*N*_*i*+1_)(*N*_*i*_+*N*_*i*+2_)+(*N*_*r*−1_*N*_*r*_)[(*N*_*r*−2_+*N*_*r*_)(*N*_*r*−1_+*N*)]+(*N*_*r*_*N*)[(*N*_*r*−1_+*N*)(*N*_*r*_)]
- *PM*_1_(*Z*)=(*N*_1_+*M*+*N*_2_)^*MN*_1_^ × (*M*+*N*_2_+*N*_1_+*N*_3_)^*N*_1_*N*_2_^ × ∏_*i*=2_^*r*−2^(*N*_*i*−1_+*N*_*i*+1_+*N*_*i*_+*N*_*i*+2_)^*N*_*i*_*N*_*i*+1_^ × (*N*_*r*−2_+*N*_*r*_+*N*_*r*−1_+*N*)^*N*_*r*−1_*N*_*r*_^ × (*N*_*r*−1_+*N*+*N*_*r*_)^*N*_*r*_*N*^
- *PM*_2_(*Z*)=[(*N*_1_)(*M*+*N*_2_)]^*MN*_1_^ × [(*M*+*N*_2_)(*N*_1_+*N*_3_)]^*N*_1_*N*_2_^ × ∏_*i*=2_^*r*−2^[(*N*_*i*−1_+*N*_*i*+1_)(*N*_*i*_+*N*_*i*+2_)]^*N*_*i*_*N*_*i*+1_^ × [(*N*_*r*−2_+*N*_*r*_)(*N*_*r*−1_+*N*)]^*N*_*r*−1_*N*_*r*_^ × [(*N*_*r*−1_+*N*)(*N*_*r*_)]^*N*_*r*_*N*^

**Proof**

To compute the topological indices of DNN, we use the edge partition method. In Table 1, we have calculated the degrees of end-nodes of each edge for*DNN*(*N*_1_*N*_2_ … *N*_*r*_). Now, by using the definitions and values from Table 1, we have the following results:

(i) $M_{1}\left(Z\right)=\underset{uv\in E}{\sum }\begin{array}{c} \left[d_{u}+d_{v}\right] \end{array}=\underset{uv\in E\left(N_{1},M+N_{2}\right)}{\sum }\left[d_{u}+d_{v}\right]+\underset{uv\in E\left(M+N_{2},N_{1}{+N}_{3}\right)}{\sum }\left[d_{u}+d_{v}\right]+\underset{uv\in E\left(N_{1}+N_{3},N_{2}+N_{4}\right)}{\sum }\left[d_{u}+d_{v}\right]\ldots \underset{uv\in E\left(N_{r-3}{+N}_{r-1}{,N}_{r-2}{+N}_{r}\right)}{\sum }\left[d_{u}+d_{v}\right]+\underset{uv\in E\left(\left(N_{r-2}+N_{r}N_{r-1}+N\right)\right)}{\sum }\left[d_{u}+d_{v}\right]+\underset{uv\in E\left(N_{r-1}+N,N_{r}\right)}{\sum }\left[d_{u}+d_{v}\right]$
Substituting values from Table 1, we get
  (15) $$\begin{array}{c} M_{1}\left(Z\right)=\left(MN_{1}\right)\left(N_{1}+M+N_{2}\right)+\left(N_{1}N_{2}\right)\left(M+N_{2}+N_{1}{+N}_{3}\right)+\left(N_{2}N_{3}\right)\left(N_{1}+N_{3}+N_{2}+N_{4}\right)+\ldots +\left(N_{r-2}N_{r-1}\right)\left(N_{r-3}{+N}_{r-1}{+N}_{r-2}{+N}_{r}\right)+\left(N_{r-1}N_{r}\right)\left(N_{r-2}+N_{r}{+N}_{r-1}+N\right)+\left(N_{r}N\right)\left(N_{r-1}+N+N_{r}\right)\text{.} \end{array}$$
It can be expressed as follows:
  (16) $$\begin{array}{c} M_{1}\left(Z\right)=\left(MN_{1}\right)\left(N_{1}+M+N_{2}\right)+\left(N_{1}N_{2}\right)\left(M+N_{2}+N_{1}{+N}_{3}\right)+\overset{r-2}{\underset{i=2}{\sum }}N_{i}N_{i+1}\left(N_{i-1}+N_{i+1}+N_{i}+N_{i+2}\right)+\left(N_{r-1}N_{r}\right)\left(N_{r-2}+N_{r}{+N}_{r-1}+N\right)+\left(N_{r}N\right)\left(N_{r-1}+N+N_{r}\right)\text{.} \end{array}$$
(ii) $M_{2}\left(Z\right)=\underset{uv\in E}{\sum }\begin{array}{c} \left[d_{u}d_{v}\right] \end{array}=\underset{uv\in E\left(N_{1},M+N_{2}\right)}{\sum }\left[d_{u}d_{v}\right]+\underset{uv\in E\left(M+N_{2},N_{1}{+N}_{3}\right)}{\sum }\left[d_{u}d_{v}\right]+\underset{uv\in E\left(N_{1}+N_{3},N_{2}+N_{4}\right)}{\sum }\left[d_{u}d_{v}\right]\ldots \underset{uv\in E\left(N_{r-3}{+N}_{r-1}{,N}_{r-2}{+N}_{r}\right)}{\sum }\left[d_{u}d_{v}\right]+\underset{uv\in E\left(\left(N_{r-2}+N_{r}N_{r-1}+N\right)\right)}{\sum }\left[d_{u}d_{v}\right]+\underset{uv\in E\left(N_{r-1}+N,N_{r}\right)}{\sum }\left[d_{u}d_{v}\right]$
Substituting values from Table 1, we have
  (17) $$\begin{array}{c} M_{2}\left(Z\right)=\left(MN_{1}\right)\left[\left(N_{1}\right)\left(M{+N}_{2}\right)\right]+\left(N_{1}N_{2}\right)\left[\left(M{+N}_{2}\right)\left(N_{1}+N_{3}\right)\right]+\left(N_{2}N_{3}\right)\left[\left(N_{1}+N_{3}\right)\left(N_{2}+N_{4}\right)\right]+\ldots +\left(N_{r-2}N_{r-1}\right)\left[\left(N_{r-3}+N_{r-1}\right)\left(N_{r-2}+N_{r}\right)\right]+\left(N_{r-1}N_{r}\right)\left[\left(N_{r-2}+N_{r}\right)\left(N_{r-1}+N\right)\right]+\left(N_{r}N\right)\left[\left(N_{r-1}+N\right)\left(N_{r}\right)\right]\text{.} \end{array}$$
It can be expressed as follows:
  (18) $$\begin{array}{c} M_{2}\left(Z\right)=\left(MN_{1}\right)\left[\left(N_{1}\right)\left(M{+N}_{2}\right)\right]+\left(N_{1}N_{2}\right)\left[\left(M{+N}_{2}\right)\left(N_{1}+N_{3}\right)\right]+\overset{r-2}{\underset{i=2}{\sum }}N_{i}N_{i+1}\left(N_{i-1}+N_{i+1}\right)\left(N_{i}{+N}_{i+2}\right)+\left(N_{r-1}N_{r}\right)\left[\left(N_{r-2}+N_{r}\right)\left(N_{r-1}+N\right)\right]+\left(N_{r}N\right)\left[\left(N_{r-1}+N\right)\left(N_{r}\right)\right]\text{.} \end{array}$$
(iii) *PM*_1_(*Z*)=∏_*uv*∈*E*_[*d*_*u*_+*d*_*v*_]=∏_*uv*∈*E*(*N*_1_, *M*+*N*_2_)_[*d*_*u*_+*d*_*v*_]+∏_*uv*∈*E*(*M*+*N*_2_, *N*_1_+*N*_3_)_[*d*_*u*_+*d*_*v*_]+∏_*uv*∈*E*(*N*_1_+*N*_3_, *N*_2_+*N*_4_)_[*d*_*u*_+*d*_*v*_] … +∏_*uv*∈*E*(*N*_*r*−3_+*N*_*r*−1_,*N*_*r*−2_+*N*_*r*_)_[*d*_*u*_+*d*_*v*_]+∏_*uv*∈*E*(*N*_*r*−2_+*N*_*r*_, *N*_*r*−1_+*N*)_[*d*_*u*_+*d*_*v*_]+∏_*uv*∈*E*(*N*_*r*−1_+*N*, *N*_*r*_)_[*d*_*u*_+*d*_*v*_]
Using Table 1, we get
  (19) $$\begin{array}{c} {PM}_{1}\left(Z\right)={\left(N_{1}+M+N_{2}\right)}^{MN_{1}}\times {\left(M+N_{2}+N_{1}{+N}_{3}\right)}^{N_{1}N_{2}}\times {\left(N_{1}+N_{3}+N_{2}+N_{4}\right)}^{N_{2}N_{3}}\times \ldots +{\left(N_{r-3}{+N}_{r-1}{+N}_{r-2}{+N}_{r}\right)}^{N_{r-2}N_{r-1}}\times {\left(N_{r-2}+N_{r}+N_{r-1}+N\right)}^{N_{r-1}N_{r}}\times {\left(N_{r-1}+N+N_{r}\right)}^{N_{r}N}\text{.} \end{array}$$
This can be further summarized as follows:
  (20) $$\begin{array}{c} {PM}_{1}\left(Z\right)={\left(N_{1}+M+N_{2}\right)}^{MN_{1}}\times {\left(M+N_{2}+N_{1}{+N}_{3}\right)}^{N_{1}N_{2}}\times \overset{r-2}{\underset{i=2}{\prod }}{\left(N_{i-1}+N_{i+1}+N_{i}+N_{i+2}\right)}^{N_{i}N_{i+1}}\times {\left(N_{r-2}+N_{r}{+N}_{r-1}+N\right)}^{N_{r-1}N_{r}}\times {\left(N_{r-1}+N+N_{r}\right)}^{N_{r}N}\text{.} \end{array}$$
(iv) We know, from equation (6), *PM*_2_(*Z*)=∏_*uv*∈*E*_[*d*_*u*_ × *d*_*v*_]

(21) $$\begin{array}{c} =\underset{uv\in E\left(N_{1},M+N_{2}\right)}{\prod }\left[d_{u}\times d_{v}\right]+\underset{uv\in E\left(M+N_{2},N_{1}{+N}_{3}\right)}{\prod }\left[d_{u}\times d_{v}\right]+\underset{uv\in E\left(N_{1}+N_{3},N_{2}+N_{4}\right)}{\prod }\left[d_{u}\times d_{v}\right]+\ldots +\underset{uv\in E\left(N_{r-3}{+N}_{r-1}{,N}_{r-2}{+N}_{r}\right)}{\prod }\left[d_{u}\times d_{v}\right]+\underset{uv\in E\left(N_{r-2}+N_{r}N_{r-1}+N\right)}{\prod }\left[d_{u}\times d_{v}\right]+\underset{uv\in E\left(N_{r-1}+N,N_{r}\right)}{\prod }\left[d_{u}\times d_{v}\right]\text{.} \end{array}$$

Substituting the values from Table 1, we getpi

(22) $$\begin{array}{c} {PM}_{2}\left(Z\right)={\left[\left(N_{1}\right)\left(M+N_{2}\right)\right]}^{MN_{1}}\times {\left[\left(M+N_{2}\right)\left(N_{1}{+N}_{3}\right)\right]}^{N_{1}N_{2}}\times {\left[\left(N_{1}+N_{3}\right)\left(N_{2}+N_{4}\right)\right]}^{N_{2}N_{3}}\times \ldots \times {\left[\left(N_{r-3}{+N}_{r-1}\right)\left(N_{r-2}{+N}_{r}\right)\right]}^{N_{r-2}N_{r-1}}\times {\left[\left(N_{r-2}+N_{r}\right)\left(N_{r-1}+N\right)\right]}^{N_{r-1}N_{r}}\times {\left[\left(N_{r-1}+N\right)\left(N_{r}\right)\right]}^{N_{r}N}\text{.} \end{array}$$

The above expression can be expressed as follows:

(23) $$\begin{array}{c} {PM}_{2}\left(Z\right)={\left[\left(N_{1}\right)\left(M+N_{2}\right)\right]}^{MN_{1}}\times {\left[\left(M+N_{2}\right)\left(N_{1}{+N}_{3}\right)\right]}^{N_{1}N_{2}}\times \overset{r-2}{\underset{i=2}{\prod }}{\left[\left(N_{i-1}+N_{i+1}\right)\left(N_{i}+N_{i+2}\right)\right]}^{N_{i}N_{i+1}}\times {\left[\left(N_{r-2}+N_{r}\right)\left(N_{r-1}+N\right)\right]}^{N_{r-1}N_{r}}\times {\left[\left(N_{r-1}+N\right)\left(N_{r}\right)\right]}^{N_{r}N}\text{.} \end{array}$$

**Theorem 3 .**

Let *Z*≅*DNN*(*N*_1_*N*_2_ … *N*_*r*_) be a deep neural network. Then the forgotten Zagreb index(*F*(*Z*)) and hyper-Zagreb index(*HM*(*Z*)) of DNN are given as follows:

- *F*(*Z*)=(*MN*_1_)((*N*_1_)^2^+(*M*+*N*_2_)^2^)+(*N*_1_*N*_2_)((*M*+*N*_2_)^2^+(*N*_1_+*N*_3_)^2^)+∑_*i*=2_^*r*−2^*N*_*i*_*N*_*i*+1_((*N*_*i*−1_+*N*_*i*+1_)^2^+(*N*_*i*_+*N*_*i*+2_)^2^)+(*N*_*r*−1_*N*_*r*_)((*N*_*r*−2_+*N*_*r*_)^2^+(*N*_*r*−1_+*N*)^2^)+(*N*_*r*_*N*)((*N*_*r*−1_+*N*)^2^+(*N*_*r*_)^2^).
- *HM*(*Z*)=(*MN*_1_)(*N*_1_+*M*+*N*_2_)^2^+(*N*_1_*N*_2_)(*M*+*N*_2_+*N*_1_+*N*_3_)^2^+∑_*i*=2_^*r*−2^*N*_*i*_*N*_*i*+1_(*N*_*i*−1_+*N*_*i*+1_+*N*_*i*_+*N*_*i*+2_)^2^+(*N*_*r*−1_*N*_*r*_)(*N*_*r*−2_+*N*_*r*_+*N*_*r*−1_+*N*)^2^+(*N*_*r*_*N*)(*N*_*r*−1_+*N*+*N*_*r*_)^2^.

**Proof**

To compute the topological indices of DNN, we use the edge partition method. In Table 1, we have calculated the degrees of end nodes of every edge for *DNN*(*N*_1_*N*_2_ … *N*_*r*_). Now, by using the definitions and values from Table 1, we get the results given below

(i) $F\left(Z\right)=\underset{uv\in E}{\sum }\begin{array}{c} \left[d_{u}^{2}+d_{v}^{2}\right] \end{array}=\sum_{uv\in E\left(N_{1},M+N_{2}\right)}\left[d_{u}^{2}+d_{v}^{2}\right]+\sum_{uv\in E\left(M+N_{2},N_{1}{+N}_{3}\right)}\left[d_{u}^{2}+d_{v}^{2}\right]+\sum_{uv\in E\left(N_{1}+N_{3},N_{2}+N_{4}\right)}\left[d_{u}^{2}+d_{v}^{2}\right]+\ldots +\sum_{uv\in E\left(N_{r-3}{+N}_{r-1}{,N}_{r-2}{+N}_{r}\right)}\left[d_{u}^{2}+d_{v}^{2}\right]+\sum_{uv\in E\left(\left(N_{r-2}+N_{r},N_{r-1}+N\right)\right)}\left[d_{u}^{2}+d_{v}^{2}\right]+\sum_{uv\in E\left(N_{r-1}+N,N_{r}\right)}\left[d_{u}^{2}+d_{v}^{2}\right]\text{.}$
Using Table 1, the above relation becomes
  (24) $$\begin{array}{c} F\left(Z\right)=\left(MN_{1}\right)\left({\left(N_{1}\right)}^{2}+{\left(M+N_{2}\right)}^{2}\right)+\left(N_{1}N_{2}\right)\left({\left(M+N_{2}\right)}^{2}+{\left(N_{1}{+N}_{3}\right)}^{2}\right)+\left(N_{2}N_{3}\right)\left({\left(N_{1}+N_{3}\right)}^{2}+{\left(N_{2}+N_{4}\right)}^{2}\right)+\ldots +\left(N_{r-2}N_{r-1}\right)\left({\left(N_{r-3}{+N}_{r-1}\right)}^{2}{+\left(N_{r-2}{+N}_{r}\right)}^{2}\right)+\left(N_{r-1}N_{r}\right)\left({\left(N_{r-2}+N_{r}\right)}^{2}+{\left(N_{r-1}+N\right)}^{2}\right)+\left(N_{r}N\right)\left({\left(N_{r-1}+N\right)}^{2}+{\left(N_{r}\right)}^{2}\right)\text{.} \end{array}$$
This can be summarized as follows:
  (25) $$\begin{array}{c} F\left(Z\right)=\left(MN_{1}\right)\left({\left(N_{1}\right)}^{2}+{\left(M+N_{2}\right)}^{2}\right)+\left(N_{1}N_{2}\right)\left({\left(M+N_{2}\right)}^{2}+{\left(N_{1}{+N}_{3}\right)}^{2}\right)+\overset{r-2}{\underset{i=2}{\sum }}N_{i}N_{i+1}\left({\left(N_{i-1}+N_{i+1}\right)}^{2}+{\left(N_{i}+N_{i+2}\right)}^{2}\right)+\left(N_{r-1}N_{r}\right)\left({\left(N_{r-2}+N_{r}\right)}^{2}+{\left(N_{r-1}+N\right)}^{2}\right)+\left(N_{r}N\right)\left({\left(N_{r-1}+N\right)}^{2}+{\left(N_{r}\right)}^{2}\right) \end{array}$$
(ii) $HM\left(Z\right)=\underset{uv\in E\;}{\sum }{\left(d_{u}{+d}_{v}\right)}^{2}=\underset{uv\in E\left(N_{1},M+N_{2}\right)}{\sum }{\left[d_{u}+d_{v}\right]}^{2}+\underset{uv\in E\left(M+N_{2},N_{1}{+N}_{3}\right)}{\sum }{\left[d_{u}+d_{v}\right]}^{2}+\underset{uv\in E\left(N_{1}+N_{3},N_{2}+N_{4}\right)}{\sum }{\left[d_{u}+d_{v}\right]}^{2}+\ldots +\underset{uv\in E\left(N_{r-3}{+N}_{r-1}{,N}_{r-2}{+N}_{r}\right)}{\sum }{\left[d_{u}+d_{v}\right]}^{2}+\underset{uv\in E\left(\left(N_{r-2}+N_{r}N_{r-1}+N\right)\right)}{\sum }{\left[d_{u}+d_{v}\right]}^{2}+\underset{uv\in E\left(N_{r-1}+N,N_{r}\right)}{\sum }{\left[d_{u}+d_{v}\right]}^{2}$

Substituting values from Table 1, we get

(26) $$\begin{array}{c} HM\left(Z\right)=\left(MN_{1}\right){\left(N_{1}+M+N_{2}\right)}^{2}+\left(N_{1}N_{2}\right){\left(M+N_{2}+N_{1}{+N}_{3}\right)}^{2}+\left(N_{2}N_{3}\right){\left(N_{1}+N_{3}+N_{2}+N_{4}\right)}^{2}+\ldots +\left(N_{r-2}N_{r-1}\right){\left(N_{r-3}{+N}_{r-1}{+N}_{r-2}{+N}_{r}\right)}^{2}+\left(N_{r-1}N_{r}\right){\left(N_{r-2}+N_{r}{+N}_{r-1}+N\right)}^{2}+\left(N_{r}N\right){\left(N_{r-1}+N+N_{r}\right)}^{2}\text{.} \end{array}$$

The above expression can be further summarized as follows:

(27) $$\begin{array}{c} HM\left(Z\right)=\left(MN_{1}\right){\left(N_{1}+M+N_{2}\right)}^{2}+\left(N_{1}N_{2}\right){\left(M+N_{2}+N_{1}{+N}_{3}\right)}^{2}+\overset{r-2}{\underset{i=2}{\sum }}N_{i}N_{i+1}{\left(N_{i-1}+N_{i+1}+N_{i}+N_{i+2}\right)}^{2}+\left(N_{r-1}N_{r}\right){\left(N_{r-2}+N_{r}{+N}_{r-1}+N\right)}^{2}+\left(N_{r}N\right){\left(N_{r-1}+N+N_{r}\right)}^{2}\text{.} \end{array}$$

**Theorem 4 .**

Let *Z*≅*DNN*(*N*_1_*N*_2_ … *N*_*r*_) be a deep neural network. The atom-bond connectivity index (*ABC*(*Z*)), geometric-arithmetic index (*GA*(*Z*)), sum connectivity index (*SCI*(*Z*)), and augmented Zagreb index (*AZI*(*Z*)) of DNN are given as follows:

- $ABC\left(Z\right)=\left(MN_{1}\right)\sqrt{\left(N_{1}+M+N_{2}-2\right)/\left(N_{1}\right)\left(M{+N}_{2}\right)}+\left(N_{1}N_{2}\right)\sqrt{\left(M+N_{2}+N_{1}{+N}_{3}-2\right)\left(M+N_{2}+N_{1}{+N}_{3}-2\right)/\left(M{+N}_{2}\right)\left(N_{1}+N_{3}\right)}+\sum_{i=2}^{r-2}N_{i}N_{i+1}+\left(N_{r-1}N_{r}\right)\sqrt{\left(N_{r-2}+N_{r}{+N}_{r-1}+N-2\right)/\left(N_{r-2}+N_{r}\right)\left(N_{r-1}+N\right)}+\left(N_{r}N\right)\sqrt{\left(\left(N_{r-1}+N+N_{r}-2\right)/\left(N_{r-1}+N\right)\left(N_{r}\right)\right)}$
- $GA\left(Z\right)=2\left(MN_{1}\right)\sqrt{\left(\left(N_{1}\right)\left(M{+N}_{2}\right)/\left(N_{1}+M{+N}_{2}\right)\right)}+2\left(N_{1}N_{2}\right)\sqrt{\left(\left(M{+N}_{2}\right)\left(N_{1}+N_{3}\right)/\left(M{+N}_{2}+N_{1}+N_{3}\right)\right)}+2\sum_{i=2}^{r-2}N_{i}N_{i+1}\sqrt{\left(\left(N_{i-1}+N_{i+1}\right)\left(N_{i}{+N}_{i+2}\right)/\left(N_{i-1}+N_{i+1}+N_{i}+N_{i+2}\right)\right)}+2\left(N_{r-1}N_{r}\right)\sqrt{\left(\left(N_{r-2}+N_{r}\right)\left(N_{r-1}+N-2\right)/\left(N_{r-2}+N_{r}+N_{r-1}+N\right)\right)}+2\left(N_{r}N\right)\sqrt{\left(\left(N_{r-1}+N\right)\left(N_{r}-2\right)/\left(N_{r-1}+N{+N}_{r}\right)\right)}$
- $SCI\left(Z\right)=\left(\left(MN_{1}\right)/\sqrt{\left(N_{1}\right)+\left(M+N_{2}\right)}\right)+\left(\left(N_{1}N_{2}\right)/\sqrt{\left(M+N_{2}\right)+\left(N_{1}{+N}_{3}\right)}\right)+\left(\sum_{i=2}^{r-2}N_{i}N_{i+1}/{\left(\left(N_{i-1}+N_{i+1}\right)+\left(N_{i}+N_{i+2}\right)\right)}^{\left(1/2\right)}\right)+\left(\left(N_{r-1}N_{r}\right)/\sqrt{\left(N_{r-2}+N_{r}\right)\left(N_{r-1}+N\right)}\right)+\left(\left(N_{r}N\right)/\sqrt{\left(N_{r-1}+N\right)+\left(N_{r}\right)}\right)$
- *AZI*(*Z*)=(*MN*_1_)((*N*_1_)(*M*+*N*_2_)/*N*_1_+*M*+*N*_2_)^3^+(*N*_1_*N*_2_)((*M*+*N*_2_)(*N*_1_+*N*_3_)/*M*+*N*_2_+*N*_1_+*N*_3_)^3^+∑_*i*=2_^*r*−2^*N*_*i*_*N*_*i*+1_((*N*_*i*−1_+*N*_*i*+1_)(*N*_*i*_+*N*_*i*+2_)/*N*_*i*−1_+*N*_*i*+1_+*N*_*i*_+*N*_*i*+2_)^3^+(*N*_*r*−1_*N*_*r*_)((*N*_*r*−2_+*N*_*r*_)(*N*_*r*−1_+*N*)/*N*_*r*−2_+*N*_*r*_+*N*_*r*−1_+*N*)^3^+(*N*_*r*_*N*)((*N*_*r*−1_+*N*)(*N*_*r*_ − 2)/*N*_*r*−1_+*N*+*N*_*r*_)^3^

**Proof**

(i)
$ABC\left(Z\right)=\sum_{uv\in E}\sqrt{\left(d_{u}+d_{v}-2/d_{u}d_{v}\right)}=\sum_{uv\in E\left(N_{1},M+N_{2}\right)}\sqrt{\left(d_{u}+d_{v}-2/d_{u}d_{v}\right)}+\sum_{uv\in E\left(M+N_{2},N_{1}{+N}_{3}\right)}\sqrt{\left(d_{u}+d_{v}-2/d_{u}d_{v}\right)}+\sum_{uv\in E\left(N_{1}+N_{3},N_{2}+N_{4}\right)}\sqrt{\left(d_{u}+d_{v}-2/d_{u}d_{v}\right)}+\ldots +\sum_{uv\in E\left(N_{r-3}{+N}_{r-1}{,N}_{r-2}{+N}_{r}\right)}\sqrt{\left(d_{u}+d_{v}-2/d_{u}d_{v}\right)}+\sum_{uv\in E\left(N_{r-2}+N_{r},N_{r-1}+N\right)}\sqrt{\left(d_{u}+d_{v}-2/d_{u}d_{v}\right)}+\sum_{uv\in E\left(N_{r-1}+N,N_{r}\right)}\sqrt{\left(d_{u}+d_{v}-2/d_{u}d_{v}\right)}$

Using edge partition in Table 1, we have
  (28) $$\begin{array}{c} ABC\left(Z\right)=\left(MN_{1}\right)\sqrt{\frac{\left(N_{1}+M+N_{2}-2\right)}{\left(N_{1}\right)\left(M{+N}_{2}\right)}}+\left(N_{1}N_{2}\right)\sqrt{\frac{\left(M+N_{2}+N_{1}{+N}_{3}-2\right)}{\left(M{+N}_{2}\right)\left(N_{1}+N_{3}\right)}}+\left(N_{2}N_{3}\right)\sqrt{\frac{\left(N_{1}+N_{3}+N_{2}+N_{4}-2\right)}{\left(N_{1}+N_{3}\right)\left(N_{2}+N_{4}\right)}}+\ldots +\left(N_{r-2}N_{r-1}\right)\sqrt{\frac{\left(N_{r-3}{+N}_{r-1}{+N}_{r-2}{+N}_{r}-2\right)}{\left(N_{r-3}+N_{r-1}\right)\left(N_{r-2}+N_{r}\right)}}+\left(N_{r-1}N_{r}\right)\sqrt{\frac{\left(N_{r-2}+N_{r}{+N}_{r-1}+N-2\right)}{\left(N_{r-2}+N_{r}\right)\left(N_{r-1}+N\right)}}+\left(N_{r}N\right)\sqrt{\frac{\left(N_{r-1}+N+N_{r}-2\right)}{\left(N_{r-1}+N\right)\left(N_{r}\right)}}\text{.} \end{array}$$
which can be shortened as follows:
  (29) $$\begin{array}{c} ABC\left(Z\right)=\left(MN_{1}\right)\sqrt{\frac{\left(N_{1}+M+N_{2}-2\right)}{\left(N_{1}\right)\left(M{+N}_{2}\right)}}+\left(N_{1}N_{2}\right)\sqrt{\frac{\left(M+N_{2}+N_{1}{+N}_{3}-2\right)}{\left(M{+N}_{2}\right)\left(N_{1}+N_{3}\right)}}+\overset{r-2}{\underset{i=2}{\sum }}N_{i}N_{i+1}\sqrt{\left(\left(N_{i-1}+N_{i+1}+N_{i}+N_{i+2}-2\right)/\left(N_{i-1}+N_{i+1}\right)\left(N_{i}+N_{i+2}\right)\right)}+\left(N_{r-1}N_{r}\right)\sqrt{\frac{\left(N_{r-2}+N_{r}{+N}_{r-1}+N-2\right)}{\left(N_{r-2}+N_{r}\right)\left(N_{r-1}+N\right)}}+\left(N_{r}N\right)\sqrt{\frac{\left(N_{r-1}+N+N_{r}-2\right)}{\left(N_{r-1}+N\right)\left(N_{r}\right)}}\text{.} \end{array}$$
(ii)
$GA\left(Z\right)=\sum_{uv\in E}\left(2\sqrt{d_{u}d_{v}}/d_{u}{+d}_{v}\right)=\sum_{uv\in E\left(N_{1},M+N_{2}\right)}\left(2\sqrt{d_{u}d_{v}}/d_{u}{+d}_{v}\right)+\sum_{uv\in E\left(M+N_{2},N_{1}{+N}_{3}\right)}\left(2\sqrt{d_{u}d_{v}}/d_{u}{+d}_{v}\right)+\sum_{uv\in E\left(N_{1}+N_{3},N_{2}+N_{4}\right)}\left(2\sqrt{d_{u}d_{v}}/d_{u}{+d}_{v}\right)+\ldots +\sum_{uv\in \left(N_{r-3}{+N}_{r-1}{,N}_{r-2}{+N}_{r}\right)}\left(2\sqrt{d_{u}d_{v}}/d_{u}{+d}_{v}\right)+\sum_{uv\in E\left(\left(N_{r-2}+N_{r},N_{r-1}+N\right)\right)}\left(2\sqrt{d_{u}d_{v}}/d_{u}{+d}_{v}\right)+\sum_{uv\in E\left(N_{r-1}+N,N_{r}\right)}\left(2\sqrt{d_{u}d_{v}}/d_{u}{+d}_{v}\right)$

Using Table 1, we get
  (30) $$\begin{array}{c} GA\left(Z\right)=2\left(MN_{1}\right)\sqrt{\frac{\left(N_{1}\right)\left(M{+N}_{2}\right)}{\left(N_{1}+M{+N}_{2}\right)}}+2\left(N_{1}N_{2}\right)\sqrt{\frac{\left(M{+N}_{2}\right)\left(N_{1}+N_{3}\right)}{\left(M{+N}_{2}+N_{1}+N_{3}\right)}}+2\left(N_{2}N_{3}\right)\sqrt{\frac{\left(N_{1}+N_{3}\right)\left(N_{2}+N_{4}\right)}{\left(N_{1}+N_{3}+N_{2}+N_{4}\right)}}+\ldots +2\left(N_{r-2}N_{r-1}\right)\sqrt{\frac{\left(N_{r-3}+N_{r-1}\right)\left(N_{r-2}+N_{r}\right)}{\left(N_{r-3}+N_{r-1}+N_{r-2}+N_{r}\right)}}+2\left(N_{r-1}N_{r}\right)\sqrt{\frac{\left(N_{r-2}+N_{r}\right)\left(N_{r-1}+N\right)}{\left(N_{r-2}+N_{r}+N_{r-1}+N\right)}}+2\left(N_{r}N\right)\sqrt{\frac{\left(N_{r-1}+N\right)\left(N_{r}\right)}{\left(N_{r-1}+N{+N}_{r}\right)}}\text{.} \end{array}$$
This can be expressed as follows:
  (31) $$\begin{array}{c} GA\left(Z\right)=2\left(MN_{1}\right)\sqrt{\frac{\left(N_{1}\right)\left(M{+N}_{2}\right)}{\left(N_{1}+M{+N}_{2}\right)}}+2\left(N_{1}N_{2}\right)\sqrt{\frac{\left(M{+N}_{2}\right)\left(N_{1}+N_{3}\right)}{\left(M{+N}_{2}+N_{1}+N_{3}\right)}}+2\overset{r-2}{\underset{i=2}{\sum }}N_{i}N_{i+1}\sqrt{\left(\left(N_{i-1}+N_{i+1}\right)\left(N_{i}{+N}_{i+2}\right)/\left(N_{i-1}+N_{i+1}+N_{i}+N_{i+2}\right)\right)}+2\left(N_{r-1}N_{r}\right)\sqrt{\frac{\left(N_{r-2}+N_{r}\right)\left(N_{r-1}+N\right)}{\left(N_{r-2}+N_{r}+N_{r-1}+N\right)}}+2\left(N_{r}N\right)\sqrt{\frac{\left(N_{r-1}+N\right)\left(N_{r}\right)}{\left(N_{r-1}+N{+N}_{r}\right)}}. \end{array}$$
(iii)
$SCI\left(Z\right)=\sum_{uv\in E}\left(1/\sqrt{d_{u}{+d}_{v}}\right)=\underset{uv\in E\left(N_{1},M+N_{2}\right)}{\sum }\left(1/\sqrt{d_{u}{+d}_{v}}\right)+\sum_{uv\in E\left(M+N_{2},N_{1}{+N}_{3}\right)}\left(1/\sqrt{d_{u}{+d}_{v}}\right)+\sum_{uv\in E\left(N_{1}+N_{3},N_{2}+N_{4}\right)}\left(1/\sqrt{d_{u}{+d}_{v}}\right)+\ldots +\sum_{uv\in E\left(N_{r-3}{+N}_{r-1}{,N}_{r-2}{+N}_{r}\right)}\left(1/\sqrt{d_{u}{+d}_{v}}\right)+\sum_{uv\in E\left(N_{r-2}+N_{r}N_{r-1}+N\right)}\left(1/\sqrt{d_{u}{+d}_{v}}\right)+\sum_{uv\in E\left(N_{r-1}+N,N_{r}\right)}\left(1/\sqrt{d_{u}{+d}_{v}}\right)$

Using Table 1, we get
  (32) $$\begin{array}{c} SCI\left(Z\right)=\frac{\left(MN_{1}\right)}{\sqrt{\left(N_{1}\right)+\left(M+N_{2}\right)}}+\frac{\left(N_{1}N_{2}\right)}{\sqrt{\left(M+N_{2}\right)+\left(N_{1}{+N}_{3}\right)}}+\frac{\left(N_{2}N_{3}\right)}{\sqrt{\left(N_{1}+N_{3}\right)+\left(N_{2}+N_{4}\right)}}+\ldots +\frac{\left(N_{r-2}N_{r-1}\right)}{\sqrt{\left(N_{r-3}{+N}_{r-1}\right)+\left(N_{r-2}{+N}_{r}\right)}}+\frac{\left(N_{r-1}N_{r}\right)}{\sqrt{\left(N_{r-2}+N_{r}\right)\left(N_{r-1}+N\right)}}+\frac{\left(N_{r}N\right)}{\sqrt{\left(N_{r-1}+N\right)+\left(N_{r}\right)}}\text{.} \end{array}$$
This can be expressed as follows:
  (33) $$\begin{array}{c} SCI\left(Z\right)=\frac{\left(MN_{1}\right)}{\sqrt{\left(N_{1}\right)+\left(M+N_{2}\right)}}+\frac{\left(N_{1}N_{2}\right)}{\sqrt{\left(M+N_{2}\right)+\left(N_{1}{+N}_{3}\right)}}+\left(\overset{r-2}{\underset{i=2}{\sum }}N_{i}N_{i+1}/{\left(\left(N_{i-1}+N_{i+1}\right)+\left(N_{i}+N_{i+2}\right)\right)}^{\left(1/2\right)}\right)+\frac{\left(N_{r-1}N_{r}\right)}{\sqrt{\left(N_{r-2}+N_{r}\right)\left(N_{r-1}+N\right)}}+\frac{\left(N_{r}N\right)}{\sqrt{\left(N_{r-1}+N\right)+\left(N_{r}\right)}}\text{.} \end{array}$$
(iv) *AZI*(*Z*)=∑_*uv*∈*E*_(*d*_*u*_×*d*_*v*_/*d*_*u*_+*d*_*v*_)^3^=∑_*uv*∈*E*(*N*_1_, *M*+*N*_2_)_(*d*_*u*_*d*_*v*_/*d*_*u*_+*d*_*v*_)^3^+∑_*uv*∈*E*(*M*+*N*_2_, *N*_1_+*N*_3_)_(*d*_*u*_*d*_*v*_/*d*_*u*_+*d*_*v*_)^3^+∑_*uv*∈*E*(*N*_1_+*N*_3_, *N*_2_+*N*_4_)_(*d*_*u*_*d*_*v*_/*d*_*u*_+*d*_*v*_)^3^+…+∑_*uv*∈(*N*_*r*−3_+*N*_*r*−1_,*N*_*r*−2_+*N*_*r*_)_(*d*_*u*_*d*_*v*_/*d*_*u*_+*d*_*v*_)^3^+∑_*uv*∈*E*((*N*_*r*−2_+*N*_*r*_*N*_*r*−1_+*N*))_(*d*_*u*_*d*_*v*_/*d*_*u*_+*d*_*v*_)^3^+∑_*uv*∈*E*(*N*_*r*−1_+*N*, *N*_*r*_)_(*d*_*u*_*d*_*v*_/*d*_*u*_+*d*_*v*_)^3^

Substituting values from Table 1, we get

(34) $$\begin{array}{c} AZI\left(Z\right)=\left(MN_{1}\right){\left(\frac{\left(N_{1}\right)\left(M{+N}_{2}\right)}{N_{1}+M{+N}_{2}}\right)}^{3}+\left(N_{1}N_{2}\right){\left(\frac{\left(M{+N}_{2}\right)\left(N_{1}+N_{3}\right)}{M{+N}_{2}+N_{1}+N_{3}}\right)}^{3}+\left(N_{2}N_{3}\right){\left(\frac{\left(N_{1}+N_{3}\right)\left(N_{2}+N_{4}\right)}{N_{1}+N_{3}+N_{2}+N_{4}}\right)}^{3}+\ldots +\left(N_{r-2}N_{r-1}\right){\left(\frac{\left(N_{r-3}{+N}_{r-1}\right)\left(N_{r-2}{+N}_{r}-2\right)}{N_{r-3}+N_{r-1}+N_{r-2}+N_{r}}\right)}^{3}+\left(N_{r-1}N_{r}\right){\left(\frac{\left(N_{r-2}+N_{r}\right)\left(N_{r-1}+N-2\right)}{N_{r-2}+N_{r}+N_{r-1}+N}\right)}^{3}+\left(N_{r}N\right){\left(\frac{\left(N_{r-1}+N\right)\left(N_{r}-2\right)}{N_{r-1}+N{+N}_{r}}\right)}^{3}\text{.} \end{array}$$

This can be abbreviated as follows:

(35) $$\begin{array}{c} AZI\left(Z\right)=\left(MN_{1}\right){\left(\frac{\left(N_{1}\right)\left(M{+N}_{2}\right)}{N_{1}+M{+N}_{2}}\right)}^{3}+\left(N_{1}N_{2}\right){\left(\frac{\left(M{+N}_{2}\right)\left(N_{1}+N_{3}\right)}{M{+N}_{2}+N_{1}+N_{3}}\right)}^{3}+\overset{r-2}{\underset{i=2}{\sum }}N_{i}N_{i+1}{\left(\frac{\left(N_{i-1}+N_{i+1}\right)\left(N_{i}{+N}_{i+2}\right)}{N_{i-1}+N_{i+1}+N_{i}+N_{i+2}}\right)}^{3}+\left(N_{r-1}N_{r}\right){\left(\frac{\left(N_{r-2}+N_{r}\right)\left(N_{r-1}+N-2\right)}{N_{r-2}+N_{r}+N_{r-1}+N}\right)}^{3}+\left(N_{r}N\right){\left(\frac{\left(N_{r-1}+N\right)\left(N_{r}-2\right)}{N_{r-1}+N{+N}_{r}}\right)}^{3}\text{.} \end{array}$$

## 4. Conclusions

The deep neural network is helpful in modeling compounds with desirable physical and chemical properties employing the structure of compounds. This paper gives computational insight into the degree-dependent topological indices, which include the Randic index, Zagreb index, multiplicative Zagreb indices, harmonic index, ABC index, GA index, and sum-connectivity index of a general DNN with r-hidden layers. These indices correlate the structure with the properties such as boiling point, molar refractivity (MR), molar volume (MV), polar surface area, surface tension, enthalpy of vaporization, flash point, and many others. The results computed in the above theorems give generally closed formulas that can be exploited to compute the topological indices of neural networks under study by giving specific values to the input parameters. The values of the computed indices grow with the growth of hidden layers and also depend on the number of nodes in each layer.

A deep neural network is an important tool used in experimental design, data reduction, fault diagnosis, and process control. The QSAR studies must be integrated with the neural network approach in order to achieve a more physical understanding of the system. The use of DNN provides an alternative way of predicting physical properties and its linkage with topological indices can further enhance theoretical achievements.

This study can be extended further by analyzing the distance-based topological indices such as the Wiener index, Harary index, and PI index. Computation of spectral invariants of deep neural networks such as energy, Estrada energy, and Kirchhoff index is also open for further research in this area.

## Figures and Tables

**Table 1:** The edge partition of *DNN*(*N*_1_*N*_2_ … *N*_*r*_) based on degrees of end nodes.

(*d*_*u*_, *d*_*v*_), *uv* ∈ *E*(*Z*)	Number of edges of the form (*d*_*u*_, *d*_*v*_)
(*N*_1_, *M*+*N*_2_)	*MN*_1_
(*M*+*N*_2_, *N*_1_+*N*_3_)	*N*_1_ *N*_2_
(*N*_1_+*N*_3_, *N*_2_+*N*_4_)	*N*_2_ *N*_3_
⋮	⋮
(*N*_*r*−3_+*N*_*r*−1_, *N*_*r*−2_+*N*_*r*_)	*N*_*r*−2_ *N*_*r*−1_
(*N*_*r*−2_+*N*_*r*_, *N*_*r*−1_+*N*)	*N*_*r*−1_ *N*_*r*_
(*N*_*r*−1_+*N*, *N*_*r*_)	*N*_*r*_ *N*

## Data Availability

No data were used to support the findings of this study.
